# The DetectDeviatingCells algorithm was a useful addition to the toolkit for cellwise error detection in observational data

**DOI:** 10.1016/j.jclinepi.2023.02.015

**Published:** 2023-02-17

**Authors:** Laura Viviani, Ian R. White, Elizabeth J. Williamson, James Carpenter, Jan van der Meulen, David A. Cromwell

**Affiliations:** a Faculty of Public Health and Policy, Department of Health Services Research and Policy, London School of Hygiene and Tropical Medicine, 15-17 Tavistock Place, London WC1H 9SH, UK; b Medical Research Council Clinical Trials Unit at University College London, 90 High Holborn, London WC1V 6LJ, UK; c Faculty of Epidemiology and Population Health, Department of Medical Statistics, London School of Hygiene and Tropical Medicine, Keppel Street, London WC1E 7HT, UK

**Keywords:** Error detection, Outlier, Data quality, DetectDeviatingCells, Mahalanobis distance, Robust statistics

## Abstract

**Objectives:**

We evaluated the error detection performance of the DetectDeviatingCells (DDC) algorithm which flags data anomalies at observation (casewise) and variable (cellwise) level in continuous variables. We compared its performance to other approaches in a simulated dataset.

**Study Design and Setting:**

We simulated height and weight data for hypothetical individuals aged 2–20 years. We changed a proportion of height values according to predetermined error patterns. We applied the DDC algorithm and other error-detection approaches (descriptive statistics, plots, fixed-threshold rules, classic, and robust Mahalanobis distance) and we compared error detection performance with sensitivity, specificity, likelihood ratios, predictive values, and receiver operating characteristic (ROC) curves.

**Results:**

At our chosen thresholds error detection specificity was excellent across all scenarios for all methods and sensitivity was higher for multivariable and robust methods. The DDC algorithm performance was similar to other robust multivariable methods. Analysis of ROC curves suggested that all methods had comparable performance for gross errors (e.g., wrong measurement unit), but the DDC algorithm outperformed the others for more complex error patterns (e.g., transcription errors that are still plausible, although extreme).

**Conclusions:**

The DDC algorithm has the potential to improve error detection processes for observational data.

## 1 Introduction and aim

Data quality controls in observational studies can be time-consuming, especially if data verification processes that involve inspection of the original data sources are required when anomalies are found during analysis. Ideally, analysts require error detection methods that correctly identify data errors. Implementing such methods is not straightforward and requires finding a trade-off between false positives (genuine values flagged as errors) and false negatives (errors not flagged as such).

Common error detection techniques include visual plots, computation of descriptive statistics, and measures based on distances such as Mahalanobis [1]. Univariable methods have the advantage of being straightforward to implement but tend to identify fewer data anomalies than multivariable approaches, which exploit all information given by the multivariable correlation structure. One disadvantage of multivariable approaches is that they often flag an error at an observation level, without specifying in which variable the error is likely to be present.

Rousseeuw and Van den Bossche [2] devised an algorithm, DetectDeviatingCells (DDC), that flags data anomalies for continuous variables; it differs from commonly used multivariable approaches because it flags cellwise outliers, i.e., values of a variable within an individual, or unit’s data (a cell) in addition to rowwise outliers (an observation). The DDC is implemented in commonly used statistical software, such as R [3], making it easily accessible to analysts for data cleaning processes.

The aim of this article is to carefully explore the performance of the DDC algorithm compared to other data checking approaches in observational data by simulating a realistic dataset of growth data into which we introduced different patterns of data errors.

## 2 Statistical methods

### 2.1 Simulated dataset and introduction of errors

We created a simulated dataset of growth data mimicking 5,000 records for individuals aged 2–20 years, containing data on age- and sex-specific height and weight values. We generated these four variables using the Centers for Disease Control and Prevention (CDC) 2000 growth charts [4], available online at https://www.cdc.gov/growthcharts/percentile_data_files.htm.

The CDC charts provide age and sex specific parameters to generate height and weight values. We generated data with equal numbers in each group of age (months) and sex as outlined in the charts. For each age/sex group, we randomly drew Z values from a standard normal distribution, and we computed height measurements according to the Box-Cox transformation [5], provided by the equation:

$$\;height\;=M{\left(1+LSZ\right)}^{\left(1/L\right)}$$

where L, M, and S are the age- and sex-specific power (L ≠ 0), median and generalized coefficient of variation.

Prevalence of errors in observational data varies across settings and this information is usually not publicly available. For this reason, we set these two levels of prevalence in our simulations following our personal experience from data management in observational studies to reflect a range of plausible settings with low and high levels of data contamination by errors. We then contaminated some randomly chosen height values according to different scenarios. In the first set of scenarios, 10% of randomly selected height measurements were altered according to four different patterns of data errors; in the second set, 2% of randomly selected heights were altered. The four error patterns we used (described below) were chosen to mimic plausible processes that could happen in clinical practice, such as transcription errors and anomalies generated by measurement instrument miscalibration. We introduced errors in one variable only to limit the complexity of the scenarios for evaluation purposes.

We devised the following four error patterns, corresponding to different levels of detection difficulty:

1. Skip last digit: we deleted the last digit of height measurements;
2. Swap last digits: we swapped the last 2 digits of height measurements;
3. Add 40 cm: we increased the original height measurement by 40 cm, so the errors corresponded to a shift in location;
4. Sample from first percentile: we replaced height values from randomly sampled draws from a truncated Normal distribution from below the first percentile of age-specific height measurements; this would yield plausible (albeit “extreme”) values of height for a given age, but these values would be relatively implausible once weight is taken into consideration.

The first two error patterns reflect transcription errors that can easily happen in practice. The third error pattern mimics errors such as mis-calibration of the measurement instrument and unit conversion mistakes. We chose to increase values by 40 cm because this was approximately 1.5 standard deviations of the height distribution without errors and, from visual plots, was deemed an acceptable level of detection difficulty for the purposes of our study. The fourth error pattern reflects a failure to comply with patients’ inclusion criteria where, for example, patients with medical conditions affecting growth have been included along with healthy controls, resulting in a mixture distribution of height (or weight).

### 2.2 Error detection methods

We applied the DDC algorithm and other error checking techniques for continuous variables: robust standard deviation scores (SDS), boxplots, bagplots [6], and classical [1] and robust versions [7–9] of the Mahalanobis distance. We excluded from the analysis the dichotomous variable sex to mimic the unmeasurable variability in the data.

**Fig. 1:**
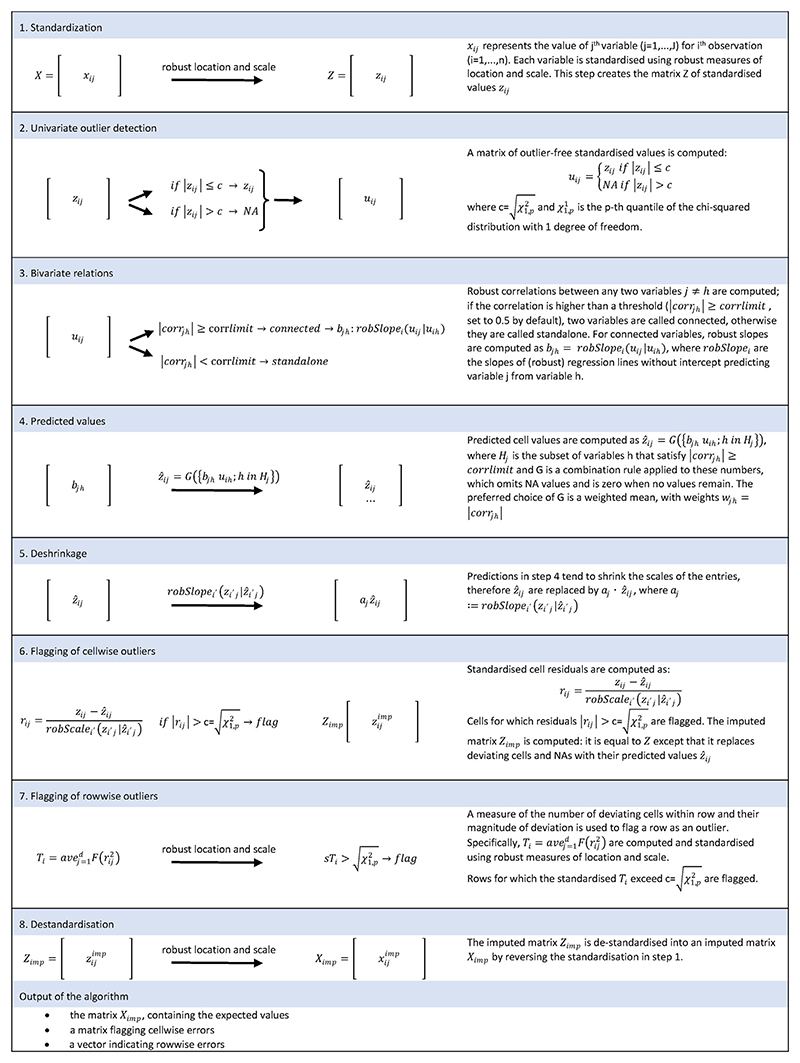
Outline of the DetectDeviatingCell algorithm.

The DDC algorithm, as outlined in Figure 1, computes robust pairwise correlations between variables and computes expected values for each data cell using the information from pairs of variables that exceed a predefined threshold of correlation. Then, the algorithm flags potential cellwise outliers when robust standardized residuals exceed another fixed threshold and flags rowwise (i.e., patient) outliers when the number and magnitude of cellwise outliers within a row (patient’s data) exceed a third fixed threshold (for more detail, see Online Supplement 1). The error detection performance can be tuned by modifying these three thresholds.

For the SDS error detection approach, for each variable, we computed a robust version of the SDS by subtracting the median from each value and then dividing by the median absolute deviation (MAD) multiplied by 1.4826 (corresponding to a robust estimate of the standard deviation). We finally classified as potential outliers cells whose SDS values were outside the (–2; 2) interval.

Boxplots flag cellwise outliers by identifying values that fall outside the range defined by (*Q*_1_ − 1.5× *IQR*; *Q*_3_ +1.5× *IQR*), where *Q*_1_ and *Q*_3_ are the first and third quartiles and *IQR* is the inter-quartile range.

Bagplots are a bivariate extension of boxplots proposed by Rousseeuw et al. [6]; they use the halfspace depth proposed by Tukey [10] to plot the “bag”, an area containing at most 50% of the data points, and an outer ring called the “fence,” obtained by inflating the bag by a factor (usually 3); values outside the fence are flagged as outliers. Online supplement 2 shows an example of a bagplot for the data used in this study.

We also applied three multivariable methods based on distances, which flag rowwise outliers by identifying observations that exceed a fixed multi-dimensional distance from the centroid of the data. The first was the classical Mahalanobis distance [1]. However, this is well known to be affected by masking, a phenomenon where outliers are not detected because they can adversely affect the estimates of location and spread used to calculate the distance of an observation from the centre of the data. For this reason, we also computed two robust versions of the Mahalanobis distance: the minimum covariance determinant (MCD) algorithm [7,8] and the minimum volume ellipsoid (MVE) estimator [7,9], which use robust approaches to compute the centre of the data and its variability. Table 1 outlines the thresholds we used to classify observations as outliers and other method-specific settings.

### 2.3 Performance evaluation

We evaluated outlier detection performance by sensitivity (probability of a true error being flagged as such), specificity (probability of a genuine value not being flagged as error), positive likelihood ratios (LR)+, probability of a true error being flagged as such divided by the (probability of a genuine value being flagged as an error), and positive predictive values ([PPV] probability of a flagged error being a true error). All performance measures were computed at cell level, given we introduced errors in one variable only.

Direct comparison of method performance is challenging for two reasons. First, it is difficult to translate the threshold for outlier classification used by one method into an equivalent threshold used by another method. Second, some methods have “tuning factors” that do not have an equivalent in other methods. For example, the DDC algorithm computes expected values using variables that show a robust correlation coefficient higher than a predefined threshold (0.5 as default in the R implementation). This does not have a counterpart in the other error detection methods used in the study.

To address these problems we compared the receiver operating characteristic (ROC) curves for each method across the four patterns of data errors. This allowed us to evaluate error detection performance across different thresholds giving a more comprehensive picture of error detection performance. We also computed sensitivity and specificity associated with the cutoff defined by the Youden index [11] to further inform error detection performance across methods using a shared optimum criterion: the Youden index, defined as *J = Sensitivity* + *Specificity* – 1, is equivalent to the maximum vertical distance between the ROC curve and the diagonal line of no discrimination and it occurs at the cutoff point where the number of correctly classified individuals is maximized [12]. It is worth emphasizing that we computed the Youden index to evaluate the different methods’ error detection performance in an “equal setting” i.e., where sensitivity and specificity are equally important; in fact, each method yielded different values of sensitivity and specificity at the “default” threshold values, making the comparison of error detection performance difficult. In some settings, maximizing sensitivity might be more important than maximizing specificity, or vice versa, in which case one might wish to allocate different weights to these two components.

We carried out data management and statistical analysis with R version 4.0.3, using packages cellWise (v2.2.5; Jakob Raymaekers and Peter Rousseeuw, 2021), epiR (v2.0.26 Mark Stevenson, 2021), aplpack (v190512, Hans Peter Wolf, 2019), heplots (v1.3-9, John Fox and Michael Friendly and Georges Monette, 2021), pROC (Xavier Robin, Natacha Turck, Alexandre Hainard, Natalia Tiberti, Frédérique Lisacek, Jean-Charles Sanchez and Markus Müller, 2011).

## 3 Results

Summary statistics for the variables age, weight, and height are given in Table 2. The distribution of weight was highly skewed and we applied a log transformation to make it more symmetrical because standard boxplots work better for symmetrical data [13,14] and because the DDC algorithm performs better for normally distributed data [2].

**Fig. 2:**
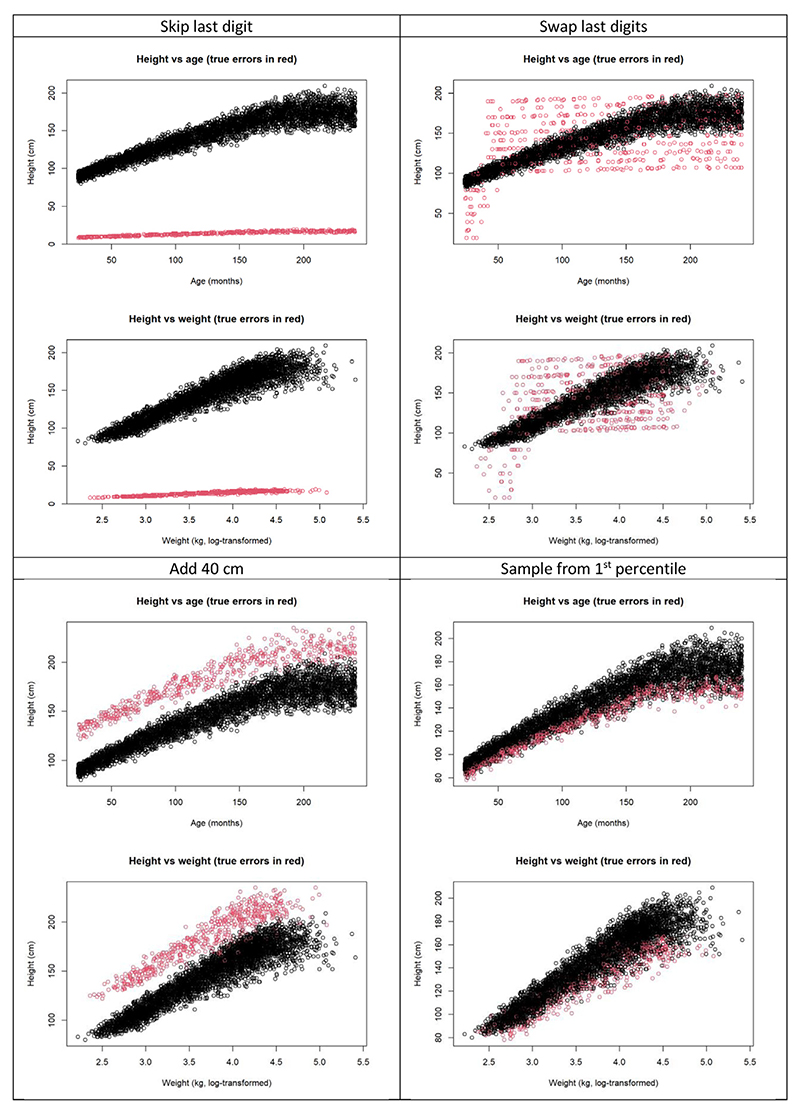
Scatterplot of height vs. age and height vs. (log-transformed) weight, for each error pattern (error prevalence 10%). Errors are in red.

Figure 2 shows scatterplots of height vs. age and weight (with error-free values shown in black), for each error pattern. The plots highlight how the variables age, height, and weight are highly correlated. They also illustrate the different challenges posed by the error patterns: some errors (e.g., “skip last digit”) are easily detected by visual inspection and summary statistics (Table 2), whereas others (e.g., “sample from first percentile”) are not.

**Fig. 3:**
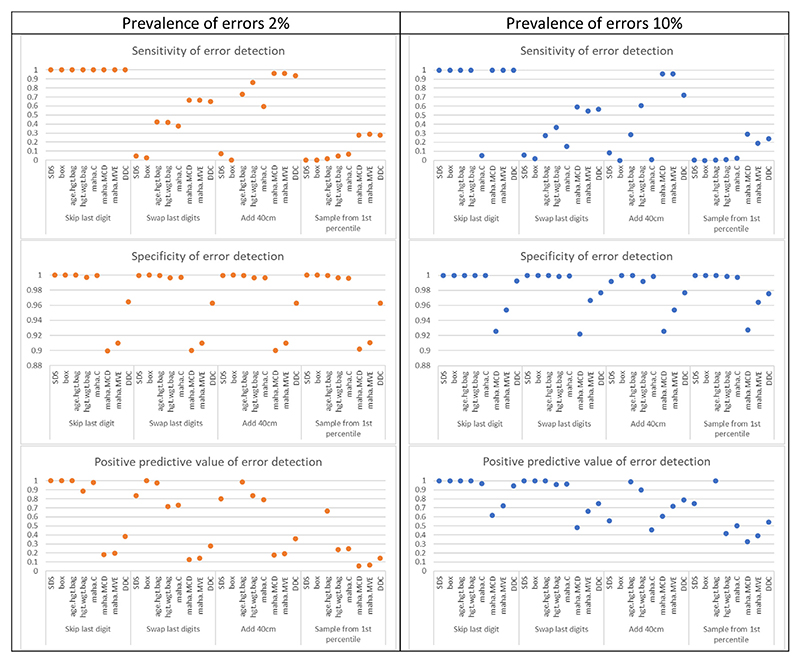
Performance measures for error detection for variable height according to different error detection approaches using thresholds in Table 1. *Abbreviations*: SDS, robust standard deviation scores; box, boxplot; age.hgt.bag, bagplot of age vs. height; hgt.wgt.bag, bagplot of height vs. weight; maha.C, classic Mahalanobis distance; maha.MCD, Mahalanobis distance computed using the MCD approach; maha.MVE, Mahalanobis distance computed using the MVE approach; DDC, DetectDeviatingCells algorithm.

Sensitivity of error detection varied considerably across methods and scenarios. In the set of scenarios where error prevalence was 2%, all methods correctly flagged as errors all values where the last digit was skipped (Fig. 3, Supplement 3.1). For the other error patterns, the univariable methods (SDS, boxplots) performed worse than multivariable methods (bagplots, Mahalanobis distance, DDC): sensitivity was at most 4.6% (95% confidence interval [CI]: 1.5, 10.5) for SDS and 2.8% (95% CI: 0.6, 7.9) for boxplots. Overall, the DDC algorithm had a comparable performance to the two robust Mahalanobis distance methods across the different patterns of error, being 64.8% (95% CI: 55.0, 73.8) vs. 66.7% (95% CI: 56.9, 75.4) for both MVE and MCD methods in the “swap last digits” pattern, and 93.5% (95% CI: 87.1; 97.4) vs. 96.3% (95% CI: 90.8; 99.0) for both MVE and MCD methods in the “add 40 cm” pattern.

For all methods, the worst performance was for the “sample from below first percentile” pattern. The DDC algorithm and the robust Mahalanobis distance methods performed best, but the sensitivity of each method was very low: 28.7% for the MVE approach (95% CI: 20.4, 38.2) and 27.8% (95% CI: 19.6, 37.2) for DDC and MCD approaches.

The results from the scenarios with 10% error prevalence mirrored the results we described for error prevalence 2% (Figure 3, Supplement 3.2). The classical Mahalanobis distance suffered from masking even in the “skip last digit” pattern, where errors could be identified by visual inspection: sensitivity was 5.4% (95% CI: 3.6, 7.6) compared to 100% (95% CI: 99.3, 100) for both MCD and MVE methods. The DDC algorithm also appeared to suffer from masking in the “add 40 cm” pattern: sensitivity dropped from 93.5% (95% CI: 87.1, 97.4) to 72.2% (95% CI: 68.2, 76.0) when we increased error prevalence from 2% to 10%.

Specificity of error detection exceeded 90% across all scenarios for all methods irrespective of error prevalence, although it was systematically lower for MVE and MCD robust Mahalanobis distance methods and DDC algorithm compared to the other approaches.

The robust MVE and MCD Mahalanobis distances and DDC algorithm yielded lower PPVs than other methods across all scenarios, although the DDC algorithm yielded higher PPVs than the MVE and MCD robust Mahalanobis distances. Overall, the PPVs were higher for the scenarios with error prevalence set to 10% than 2%.

### 3.1 Receiver Operating Characteristic curves

**Fig. 4:**
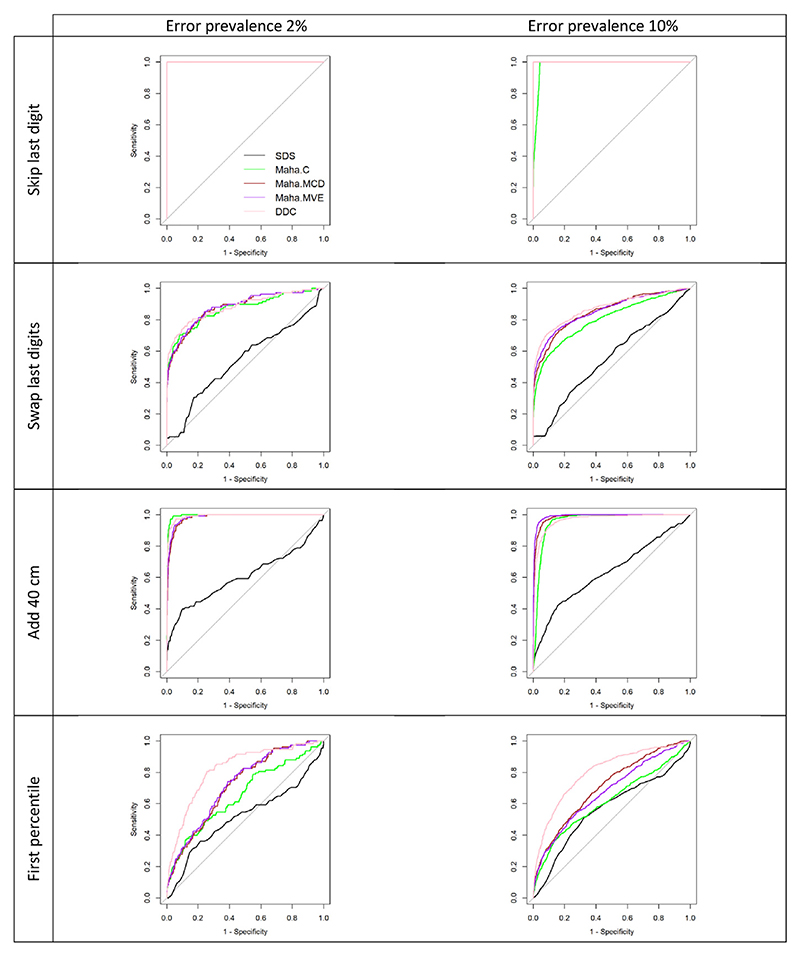
ROC analysis: ROC curves for different error detection methods, *Abbreviations*: SDS, robust standard deviation scores; Maha.C, classic Mahalanobis distance; Maha.MCD, Mahalanobis distance computed using the MCD approach; Maha.MVE, Mahalanobis distance computed using the MVE approach; DDC, DetectDeviatingCells algorithm. Note: for error “skip last digit” most of the ROC curves overlap, and are shown in pink.

Figure 4 and Table 3 show the results from the ROC analysis. All methods had comparable performance in the “skip last digit” pattern, where the ROC curves overlap. In the other error patterns, the SDS method performed poorly with a highest area under the curve (AUC) of 0.64 (95% CI: 0.61, 0.66) in the “add 40 cm” pattern with 10% error prevalence.

For the error patterns “swap last digits” and “add 40 cm”, the curves for DDC and robust Mahalanobis distances are very close, suggesting that these methods have a similar performance. The classical Mahalanobis distance performed worse than the robust version for error patterns with a 10% error prevalence. For the pattern “sample from below the first percentile,” the robust versions of the Mahalanobis distance outperformed the classical one, and the DDC algorithm performed better than the other methods in both prevalence scenarios.

When we used the same criterion for cutoff choice (Youden index), the DDC algorithm and Mahalanobis distances had comparable values of sensitivity and specificity across all scenarios (Supplement 3.3, Supplement 3.4, Supplement 3.5), and error detection performance measures showed the same pattern observed in Figure 3: robust methods performed better than classical ones; the DDC algorithm had similar performance to other robust methods; all methods performed worse in harder-to-spot scenarios (“sample from the first percentile”).

As shown in Supplement 3.6, LR + ranged from around 4 (95% CI: 3.4, 4.7 for error pattern “sample from first percentile” detected by Mahalanobis distance using MCD) to over 600 (95% CI: 158.1, 2558.4 for error “add 40 cm” detected by bagplots), without a clear pattern across scenarios.

## 4 Discussion

We found that the performance of the DDC method is similar to, or better than, commonly used error-detection methods. In particular, the ROC plots showed its performance was similar, or better, performance to other multivariable robust methods. In the harder-to-detect error patterns (such as “sample from the first percentile”), the ROC curve for the DDC algorithm was considerably higher than the other curves, and the AUC was significantly bigger, suggesting that the DDC algorithm might have a better error detection ability in circumstances when data anomalies are not so clear-cut.

We observed that nonrobust multivariable measures are prone to masking. In the scenarios with a 10% error prevalence, the classical Mahalanobis distance failed to detect errors that are easily detectable by visual inspections, such as last digit skipping. This re-enforces the common advice to include appropriate data visualization techniques as a preliminary step to data analysis, as illustrated in a seminal article by Anscombe [15] and, more recently, by the “Datasaurus Dozen” dataset [16].

As expected, robust methods performed better than classical ones, being less prone to the problem of masking; this makes a case for the use of robust measures of the centre and spread of the data when computing distances to flag potential errors. Robust approaches can be easily applied by using the median instead of the arithmetic mean and the MAD instead of the standard deviation. Boxplots can also be adapted to account for skewness in the data (see, for example, the adjusted [13] and the generalized boxplots [14], which adapt the computation of the thresholds for defining potential errors according to the expected proportion of values falling outside a range, given the underlying skewed/heavy-tailed distribution).

The DDC algorithm has some advantages over the other methods. Firstly, in comparison to other multivariable methods, it computes expected values for each data cell, and can therefore be used as a single (conditional mean) imputation method when data are missing. Further work should examine whether these expected values have the potential for the algorithm to be a data correction method. Rousseeuw and Van den Bossche [2] report that the algorithm is less efficient than the EM algorithm [17] for normally distributed data without outliers, but it is more robust when cellwise outliers are present. The evaluation of the DDC algorithm for imputation and data correction is outside the scope of this article.

Another feature of the DDC algorithm is its ability to flag errors both at a record level (rowwise) and at a data point level (cellwise). This is a highly desirable characteristic because data quality controls often require verification through clinical notes, which is a time-consuming task. The ability to flag potential errors cellwise in addition to rowwise could be a time-saving advantage, especially in big datasets.

Another advantage of the DDC algorithm is its flexibility. By modifying some parameters that are readily available in the algorithm implementation (e.g., in R), it can be tuned to use more (or less) strongly correlated variables to compute expected values (and therefore residuals, which are used to flag errors). It can also be tuned to favour sensitivity over specificity (or vice versa) by modifying the default settings in the algorithm implementation; this aspect is appropriate in settings where sensitivity and specificity do not have equal importance. However, before it can be implemented as a more or less automated error screening framework, pilot studies tailored to specific contexts are needed to tune parameters.

This study has some limitations. First, the study evaluated different methods with different tuning parameters (sometimes not comparable across methods), thus making a comparison of their performance more difficult. Moreover, the adoption of fixed thresholds, tailored to each method, further hindered comparisons. We mitigated this by carrying out an ROC analysis, which allowed us to evaluate method performance across different thresholds. We also computed the Youden index to assess sensitivity and specificity at a threshold chosen according to the same criterion.

The second limitation is that, although we simulated realistic data using CDC growth charts and included common examples of data anomalies found in clinical practice, we devised simplistic scenarios with 3 variables and errors present in only one variable according to two levels of prevalence. This allowed us to evaluate the performance of each method in a controlled environment, but the scenarios do not fully capture the complexity of most reallife processes that could lead to data errors. For this reason, we suggest that the added value of the DDC should be thoroughly explored with real data; it could be implemented after the data cleaning processes already in place, and its added detection ability could be verified through data verification procedures. This would also offer the advantage of estimating the added burden of the DDC implementation and evaluating its justification.

The DDC algorithm can be a valuable addition to the error detection toolbox for analysts, although some difficulties need to be overcome before it can be routinely used in a wide range of data quality settings. Firstly, it can be implemented only on continuous variables. It cannot use potentially explanatory information from noncontinuous variables (e.g., when there are known differences between men and women), which could improve the DDC error detection ability. This issue could be addressed in simple settings by stratifying the analysis by groups defined by the categorical variables.

Another feature that would make the DDC more adaptable to routine use is the possibility to analyze and visualize results according to group blocks of rows of varying size: for example, the DDC algorithm could be used in conjunction with heatmaps to visualize error detection patterns; the function cellMap{CellWise} in R marks in red unusually large cell values and in blue unusually low cell values. This is a useful application when data have a temporal or spatial dimension, for example with panel data where the same information is collected at different points in time. Changes in the pattern of error detection over time can be suggestive for example of change in variables definitions, or changes in measurement instruments, or issues with instrument calibration. At the moment, the cellMap function has the option to combine in one block a fixed number of rows, to be plotted in the heatmap. Expanding this option to allow this number to vary would have a wider application, for example in cases where blocks are defined by health care providers, which typically have a varying number of patients registered.

In our simulations, we evaluated the use of the DDC algorithm as an addition to the error detection toolkit, but in some scenarios (e.g., sample from the first percentile), sensitivity of error detection was low. It is worth highlighting that error detection processes consist of a set of tools that complement each other. Univariate methods such as graphical displays (e.g., histograms and boxplots) and descriptive statistics (e.g., range, high, and low percentiles) are quick and easy to implement methods to identify obvious errors in measurement units and typos; they therefore are a valuable first step in error detection processes. The DDC can be implemented as a second step, along with other multivariable methods, such as visual plots (e.g., scatterplots and mosaic plots), methods based on robust distances or methods based on regression techniques (e.g., analysis of residuals and influential statistics). Hadi et al. [18] and Hodge et al. [19] provide useful overviews of error detection methods. The choice of the most appropriate method and strategy depends on many factors: availability through commonly used software, ease of implementation, trade-off between costs and benefits, and the likely impact of errors on research output. Different methods could also be implemented iteratively in an integrated approach, but the efficiency of this strategy needs to be trialled in and tailored to specific settings.

## 5 Conclusions

Our simulation study found that use of the DDC algorithm has the potential to improve error detection processes for observational data. The implementation of the DDC algorithm incorporates a type of imputation as part of the error detection process, thereby raising the possibility of the DDC algorithm not only detecting errors, but also providing a mechanism for correcting them. Such a use of the algorithm deserves further methodological exploration.

Areas for further development of the DDC algorithm include the extension to its implementation for categorical variables and the generalization to clustering settings. Again such an extension would need further evaluation in real data with real error patterns.

## Supplementary Material

Supplementary material

## Figures and Tables

**Table 1:** Thresholds and settings for outlier classification used in this article

Method	Details	Rule for outlier classification
Robust SDS	$SDS_{rob}=\frac{x-median(x)}{MAD(x)}$ where *MAD*(*x*) = 1.4826 • *median*(|*x* – *median*(*x*)|)	SDS_rob_ < −2 or >2
Boxplot	Q1 = first quartile; Q3 = third quartile; IQR = interquartile range	Height < *Q*1 − 1.5 • *IQR* or > *Q*3 + 1.5 • *IQR*
Bagplot	Bag: an area containing at most 50% of the data points in a bivariate distribution; Fence: an outer ring, obtained by inflating the bag by a factor of 3; values outside the fence are flagged as outliers.	Point lies outside the fence
Mahalanobis distance (classic)	$\;Distance\;=\sqrt{{\left(x_{i}-{\bar{x}}_{n}\right)}^{\prime }S_{n}^{-1}\left(x_{i}-{\bar{x}}_{n}\right)}$ where ${\bar{x}}_{n}$ is the sample mean and *S_n_* the sample covariance matrix of the data.	${\text{Distance}}^{2}>\chi_{3.0.99}^{2}$
Mahalanobis distance (MCD)	Robust estimates of location and covariance are computed from an “outlier-free” subset of the data, obtained by identifying the subset of the observations with smallest determinant.	${\text{Distance}}^{2}>\chi_{3,0.99}^{2}$
Mahalanobis distance (MVE)	Robust (MVE) estimates of location and covariance are computed, by identifying the ellipsoid with minimal volume covering at least h points, with h prespecified ([n/2] + 1 ≤ h ≤ n). The Mahalanobis distance is computed using these estimates of location and covariance.	${\text{Distance}}^{2}>\chi_{3.0.99}^{2}$
DDC	The DDC algorithm consists of these steps: Computation of robust pairwise correlations between variables; Computation of expected values for each data cell using the information from pairs of variables that exceed a predefined threshold of correlation; Flagging of potential cellwise outliers when robust standardized residuals exceed another fixed threshold; Flagging of potential rowwise (i.e., patient) outliers when the number and magnitude of cellwise outliers within a row (patient’s data) exceed a third fixed threshold.	$\left|r_{ij}\right|>\text{c}=\sqrt{\chi_{1,p}^{2}}$ ^a^Other settings were: Corrlim = 0.5 tolProb = 0.99

*Abbreviations*: DDC, DetectDeviatingCells; MCD, minimum covariance determinant; MVE, minimum volume ellipsoid; SDS, standard deviation scores.

a corrlim = when computing expected values, the algorithm uses variables for which the pairwise robust correlation exceeds these thresholds; all other variables are considered standalone and treated on their own; TolProb = tolerance probability, which determines the threshold for flagging outliers in several steps of the algorithm. See Appendix for more detailed explanation of c, Corrlim and TolProb.

**Table 2:** Descriptive statistics (*n =* 5,000 observations) for age, weight (log-transformed variable), and height (cm) from the simulated dataset without errors (“error free”) and with 10% prevalence of errors in four error patterns

Statistic	Age (mo)	Weight (log kg)	Height (cm)				
			Error free	Skip last digit	Swap last digits	Add 40 cm	Sample from 1st percentile
Mean	131.5	3.7	144	130	144	149	143
SD	63.1	0.6	28.5	48.7	29.5	31.0	28.4
MAD	80.8	0.7	32.6	40.0	34.1	32.6	32.6
Minimum	23.7	2.2	80	8	19	80	78
1st Percentile	25.3	2.5	88	10	86	88	87
5th Percentile	33.9	2.7	96	14	95	96	95
10th Percentile	44.6	2.9	102	17	102	104	102
25th Percentile	76.9	3.2	121	109	120	124	120
Median	131.6	3.8	149	142	148	153	147
75th Percentile	185.8	4.2	168	166	168	172	166
90th Percentile	219.2	4.4	179	178	180	185	178
95th Percentile	230.0	4.6	184	184	186	197	184
99th Percentile	238.9	4.9	194	194	196	218	194
Maximum	240.5	5.4	209	209	209	235	209

*Abbreviations*: MAD, median absolute deviation; SD, standard deviation.

**Table 3:** Receiver operating characteristic analysis: area under the curve for different error detection methods

Method	AUC (95% CI) for error prevalence 2%			
	Skip last digit	Swap last digits	Add 40 cm	First percentile
SDS	1.000 (1.000; 1.000)	0.481 (0.542; 0.603)	0.538 (0.608; 0.677)	0.452 (0.518; 0.584)
Mahalanobis distance (classic)	1.000 (1.000; 1.000)	0.825 (0.868; 0.911)	0.992 (0.994; 0.997)	0.596 (0.654; 0.711)
Mahalanobis distance (MCD)	1.000 (1.000; 1.000)	0.840 (0.878; 0.917)	0.974 (0.981; 0.988)	0.672 (0.716; 0.761)
Mahalanobis distance (MVE)	1.000 (1.000; 1.000)	0.843 (0.881; 0.920)	0.977 (0.984; 0.990)	0.678 (0.723; 0.768)
DDC	1.000 (1.000; 1.000)	0.842 (0.883; 0.924)	0.985 (0.990; 0.995)	0.780 (0.821; 0.861)
	AUC (95% CI) for error prevalence 10%			
	Skip last digit	Swap last digits	Add 40 cm	First percentile
SDS	1.000 (1.000; 1.000)	0.552 (0.526; 0.579)	0.636 (0.609; 0.664)	0.568 (0.539; 0.596)
Mahalanobis distance (classic)	0.984 (0.981; 0.986)	0.802 (0.779; 0.825)	0.959 (0.953; 0.964)	0.618 (0.590; 0.647)
Mahalanobis distance (MCD)	1.000 (1.000; 1.000)	0.853 (0.833; 0.872)	0.985 (0.982; 0.988)	0.707 (0.684; 0.730)
Mahalanobis distance (MVE)	1.000 (1.000; 1.000)	0.857 (0.837; 0.877)	0.991 (0.989; 0.993)	0.677 (0.651; 0.702)
DDC	1.000 (1.000; 1.000)	0.873 (0.854; 0.892)	0.967 (0.960; 0.974)	0.802 (0.781; 0.822)

AUC, area under the curve; DDC, DetectDeviatingCells; MCD, the minimum covariance determinant; MVE, minimum volume ellipsoid; SDS, standard deviation scores.

## What is new?

### Key findings

- Commonly used multivariable, robust error detection methods had similar performance for gross error patterns.
- The DetectDeviatingCells algorithm outperformed these error detection methods for more complex error patterns we explored.

### What this adds to what was known?

- This finding shows that the DDC algorithm has the potential to improve error detection approaches for observational data.

### What is the implication and what should change now?

- The DDC algorithm could be added to the researcher toolkit for error detection
- The value of the DDC algorithm should be further explored with real-world data, also considering the added burden of its implementation.
