# Supplementary material for: The DetectDeviatingCells algorithm was a useful addition to the toolkit for cellwise error detection in observational data
Source: J Clin Epidemiol. Author manuscript; Available in PMC 2024 Mar 8. (PMC7615728; doi:10.1016/j.jclinepi.2023.02.015)
Supplement: Supplementary material [file EMS194376-supplement-Supplementary_material.docx]

# Online Supplements

# Online supplement 1: statistical methods

- 1. Data generating mechanism

We created a simulated dataset of growth data using the Centers for Disease Control and Prevention 2000 Growth Charts [1], available online at https://www.cdc.gov/growthcharts/percentile_data_files.htm. We generated a fixed number of age values, as defined in the CDC growth charts (from 24 to 240 months, by 0.5 months), for each sex. For each age/sex group outlined in the charts, we randomly drew values Z from a standard normal distribution, and we computed height measurements according to the equation:

height = M (1 + LSZ)**(1/L)

where L, M, and S are the power (L ≠ 0), the median (M), and the generalized coefficient of variation (S) parameters of the Box-Cox transformation [2].

We independently simulated BMI values following the same approach and we finally computed weight measurements using the simulated height and BMI measurements.

We introduced some variability to the simulated data by adding to age a value ranging from -0.5 to +0.5 months, randomly drawn from a uniform distribution and by changing height/weight values by either +10% or -10% with a probability each of 0.5.

Using the simulated values as a reference population, we finally took a random sample of n=5,000 records.

- 1. Outline of DetectDeviatingCells (DDC) algorithm

1. *Standardisation*

Each variable is standardised using robust measures of location and scale. This step creates the matrix Z of standardised values $z_{ij}$ for the jth variable in the ith person.

1. *Univariate outlier detection*

This step creates the outlier-free standardised values $u_{ij}=\left\{ \begin{matrix} z_{ij} if \left| z_{ij} \right|\leq c \\ NA if \left| z_{ij} \right|>c \end{matrix} \right.$

where the threshold value c=$\sqrt{\chi_{1,p}^{2}}$ and the default value of p in the algorithm is 99%.

1. *Bivariate relations*

This step computes robust correlations between any two variables h≠j; if the correlation is higher than a threshold (equal to 0.5 by default), two variables are called connected, otherwise they are called standalone.

The computation of the robust correlation is performed through an initial estimate, following Gnanadesikan and Kettenring [3]:

$$\hat{\rho}_{jh}=\left( \left( {robScale}_{i}\left( z_{ij}+z_{ih} \right) \right)^{2}-\left( {robScale}_{i}\left( z_{ij}-z_{ih} \right) \right)^{2} \right)/4$$

This estimate implies a tolerance ellipse with the same coverage probability p as in c=$\sqrt{\chi_{1,p}^{2}}$

The robust correlation is finally defined as the plain product-moment correlation of the data points $\left( z_{ij},z_{ih} \right)$ inside the ellipse.

For connected variables ($\left| corr \right|\geq corrlimit$), the robust slopes are computed as : $b_{jh}= {robSlope}_{i}(u_{ij}|u_{ih})$; they are the slope of the robust regression line without intercept that predicts variable j from variable h.

1. *Predicted values*

This step computes the predicted values for each cell, defined as:

$\hat{z}_{ij}=G\left( \left\{ b_{jh}u_{ih};h in H_{j} \right\} \right)$, where $H_{j}$ is the subset of variables h that satisfy $\left| corr_{jh} \right|\geq corrlimit$ and G is a combination rule applied to these numbers, which omits NA values and is zero when no values remain. The authors specified that their preferred choice of G is a weighted mean, with weights $w_{jh}=\left| {corr}_{jh} \right|$.

1. *Deshrinkage*

Predictions in step 4 tend to shrink the scales of the entries, therefore $\hat{z}_{ij}$ are replaced by ${a_{j}\cdot\hat{z}}_{ij}$, where $a_{j}:={robSlope}_{i^{'}}\left( z_{i^{'}j}|\hat{z}_{i^{'}j} \right)$

1. *Flagging cellwise outliers*

This step computes the standardised cell residuals as: $r_{ij}=\frac{z_{ij}-\hat{z}_{ij}}{{robScale}_{i^{'}}\left( z_{i^{'}j}|\hat{z}_{i^{'}j} \right)}$.

and flags the cells for which residuals exceed a threshold if $\left| r_{ij} \right|>$ c=$\sqrt{\chi_{1,p}^{2}}$.

This step produces the imputed matrix $Z_{imp}$, which is equal to $Z$ except that it replaces deviating cells and NAs with their predicted values $\hat{z}_{ij}$ (and the unflagged cells remain as they are).

The implementation in the R package offers the option of setting the flagged cells to NA and of repeating the steps 4 through 6 to improve accuracy of the estimates.

1. *Flagging rowwise outliers*

In order to flag a row as an outlier, a measure of the number of deviating cells within row and/or a measure of the magnitude of deviation can be used.

Under the null hypothesis of multivariate gaussian data without outliers, the distribution of $r_{ij}$ is close to standard Gaussian, therefore the cumulative density function of $r_{ij}^{2}$ is $\sim\chi_{1}^{2}$. Therefore, one can compute $T_{i}={ave}_{j=1}^{d}F\left( r_{ij}^{2} \right)$ and standardise it using robust measures of location and scale, and flag the rows I for which the standardised $T_{i}$ exceeds the cut-off c=$\sqrt{\chi_{1,p}^{2}}$.

1. *Destandardise*

The imputed matrix $Z_{imp}$ is de-standardised into an imputed matrix $X_{imp}$ by undoing the standardisation in step 1.

The R function “DDC” in the R package “cellwise” (v2.2.5; Jakob Raymaekers and Peter Rousseeuw, 2021) has the options corrlim, which sets the level of correlation defined at step 4 (equal to 0.5 as default) and the option tolProb (toleance probability, set to 0.99 by default), which determines the cutoff values “c” for flagging outliers used in several steps of the algorithm, as defined above.

[1] R. J. Kuczmarski, C. L. Ogden, S. S. Guo, L. M. Grummer-Strawn, K. M. Flegal, Z. Mei, R. Wei, L. R. Curtin, A. F. Roche and C. L. Johnson, 2000 CDC Growth Charts for the United States: methods and development, Series 11 ed., vol. 246, National Center for Health Statistics, 2002, pp. 1-190

[2] Box GEP, Cox DR. An analysis of transformations. J R Stat Soc, Ser B (Method) 26(2):211–34. 1964

[3] R Gnanadesikan, J.R. Kettenring. Robust Estimates, Residuals, and Outlier Detection with Multiresponse Data. Biometrics, 28: 81–124. 1972.

- 1. Receiver Operating Characteristics (ROC) analysis

Receiver Operating Characteristics (ROC) curves are a plot of sensitivity vs (1-Specificity) at increasing thresholds of status classification (e.g. disease/non-disease, error/non-error). We built empirical ROC curves for the different error detection methods by varying the observed thresholds of error classification as follows:

- SDS method: we varied the absolute values of the SDS;
- Mahalanobis distances (classical and robust): we varied the distances, as by definition they are all positive;
- DDC algorithm: we varied the absolute value of the standardised residuals computed from the DDC algorithm.

# Online supplement 2: example of bagplot


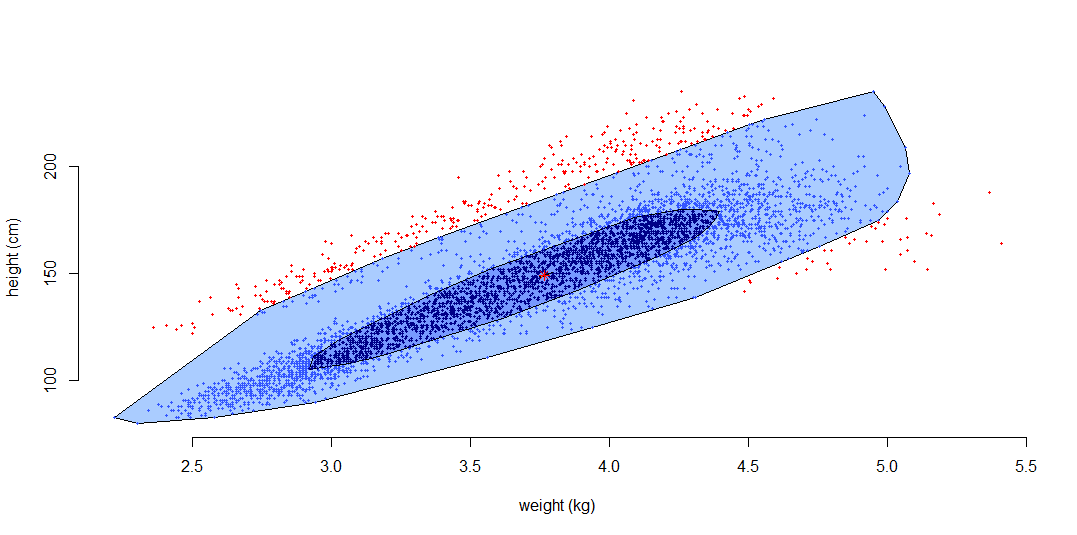


Bagplot of height vs (log-transformed) weight for the dataset with the “Add 40cm” error pattern, with 10% error prevalence. The red cross represents the depth median; the dark blue area represents the “bag” and the light blue area represent the “fence”; points lying outside the fence, coloured in red, are classified as outliers.

# Online supplement 3: measures of error detection performance

Supplement 3.1 Sensitivity, specificity and predictive values across scenarios, error prevalence 2%

| **error** | **method** | **Sensitivity**  **(95%CI)** | **Specificity**  **(95%CI)** | **PPV**  **(95%CI)** | **NPV**  **(95%CI)** |
| --- | --- | --- | --- | --- | --- |
| Skip last digit | SDS | 1.000  (0.966; 1.000) | 1.000  (0.999; 1.000) | 1.000  (0.966; 1.000) | 1.000  (0.999; 1.000) |
|  | box | 1.000  (0.966; 1.000) | 1.000  (0.999; 1.000) | 1.000  (0.966; 1.000) | 1.000  (0.999; 1.000) |
|  | age.hgt.bag | 1.000  (0.966; 1.000) | 1.000  (0.999; 1.000) | 1.000  (0.966; 1.000) | 1.000  (0.999; 1.000) |
|  | hgt.wgt.bag | 1.000  (0.966; 1.000) | 0.997  (0.995; 0.998) | 0.885  (0.815; 0.936) | 1.000  (0.999; 1.000) |
|  | maha.C | 1.000  (0.966; 1.000) | 1.000  (0.999; 1.000) | 0.982  (0.936; 0.998) | 1.000  (0.999; 1.000) |
|  | maha.MCD | 1.000  (0.966; 1.000) | 0.900  (0.891; 0.908) | 0.180  (0.150; 0.213) | 1.000  (0.999; 1.000) |
|  | maha.MVE | 1.000  (0.966; 1.000) | 0.910  (0.901; 0.918) | 0.196  (0.164; 0.232) | 1.000  (0.999; 1.000) |
|  | DDC | 1.000  (0.966; 1.000) | 0.965  (0.959; 0.970) | 0.384  (0.327; 0.444) | 1.000  (0.999; 1.000) |
| Swap last digits | SDS | 0.046  (0.015; 0.105) | 1.000  (0.999; 1.000) | 0.833  (0.359; 0.996) | 0.979  (0.975; 0.983) |
|  | box | 0.028  (0.006; 0.079) | 1.000  (0.999; 1.000) | 1.000  (0.292; 1.000) | 0.979  (0.975; 0.983) |
|  | age.hgt.bag | 0.426  (0.331; 0.525) | 1.000  0.999; 1.000) | 0.979  (0.887; 0.999) | 0.987  (0.984; 0.990) |
|  | hgt.wgt.bag | 0.417  (0.323; 0.515) | 0.996  (0.994; 0.998) | 0.714  (0.587; 0.821) | 0.987  (0.984; 0.990) |
|  | maha.C | 0.380  (0.288; 0.478) | 0.997  (0.995; 0.998) | 0.732  (0.597; 0.842) | 0.986  (0.983; 0.989) |
|  | maha.MCD | 0.667  (0.569; 0.754) | 0.900  (0.891; 0.908) | 0.128  (0.102; 0.159) | 0.992  (0.989; 0.994) |
|  | maha.MVE | 0.667  (0.569; 0.754) | 0.910  (0.901; 0.918) | 0.140  (0.111; 0.173) | 0.992  (0.989; 0.994) |
|  | DDC | 0.648  (0.550; 0.738) | 0.963  (0.957; 0.968) | 0.279  (0.224; 0.339) | 0.992  (0.989; 0.994) |
| Add 40 cm | SDS | 0.074  (0.033; 0.141) | 1.000  (0.999; 1.000) | 0.800  (0.444; 0.975) | 0.980  (0.976; 0.984) |
|  | box | 0.000  (0.000; 0.034) | 1.000  (0.999; 1.000) | - | 0.978  (0.974; 0.982) |
|  | age.hgt.bag | 0.731  (0.638; 0.812) | 1.000  (0.999; 1.000) | 0.988  (0.932; 1.000) | 0.994  (0.992; 0.996) |
|  | hgt.wgt.bag | 0.861  (0.781; 0.920) | 0.996  (0.994; 0.998) | 0.838  (0.756; 0.901) | 0.997  (0.995; 0.998) |
|  | maha.C | 0.593  (0.494; 0.686) | 0.997  (0.994; 0.998) | 0.790  (0.685; 0.873) | 0.991  (0.988; 0.993) |
|  | maha.MCD | 0.963  (0.908; 0.990) | 0.900  (0.892; 0.909) | 0.176  (0.146; 0.209) | 0.999  (0.998; 1.000) |
|  | maha.MVE | 0.963  (0.908; 0.990) | 0.910  (0.901; 0.918) | 0.190  (0.158; 0.226) | 0.999  (0.998; 1.000) |
|  | DDC | 0.935  (0.871; 0.974) | 0.963  (0.957; 0.968) | 0.357  (0.301; 0.416) | 0.999  (0.997; 0.999) |
| Sample from 1^s^t percentile | SDS | 0.000  (0.000; 0.034) | 1.000  (0.999; 1.000) | - | 0.978  (0.974; 0.982) |
|  | box | 0.000  (0.000; 0.034) | 1.000  (0.999; 1.000) | - | 0.978  (0.974; 0.982) |
|  | age.hgt.bag | 0.019  (0.002; 0.065) | 1.000  (0.999; 1.000) | 0.667  (0.094; 0.992) | 0.979  (0.974; 0.983) |
|  | hgt.wgt.bag | 0.046  (0.015; 0.105) | 0.997  (0.995; 0.998) | 0.238  (0.082; 0.472) | 0.979  (0.975; 0.983) |
|  | maha.C | 0.065  (0.026; 0.129) | 0.996  (0.993; 0.997) | 0.250  (0.107; 0.449) | 0.980  (0.975; 0.983) |
|  | maha.MCD | 0.278  (0.196; 0.372) | 0.902  (0.893; 0.910) | 0.059  (0.040; 0.083) | 0.983  (0.978; 0.986) |
|  | maha.MVE | 0.287  (0.204; 0.382) | 0.910  (0.902; 0.918) | 0.066  (0.045; 0.093) | 0.983  (0.979; 0.987) |
|  | DDC | 0.278  (0.196; 0.372) | 0.963  (0.957; 0.968) | 0.142  (0.098; 0.196) | 0.984  (0.980; 0.987) |

PPV=positive predictive value; NPV= negative predictive value

Supplement 3.2 Sensitivity, specificity and predictive values across scenarios, error prevalence 10%

| **Error** | **method** | **Sensitivity**  **(95%CI)** | **Specificity**  **(95%CI)** | **PPV**  **(95%CI)** | **NPV**  **(95%CI)** |
| --- | --- | --- | --- | --- | --- |
| Skip last digit | SDS | 1.000  (0.993; 1.000) | 1.000  (0.999; 1.000) | 1.000  (0.993; 1.000) | 1.000  (0.999; 1.000) |
|  | box | 1.000  (0.993; 1.000) | 1.000  (0.999; 1.000) | 1.000  (0.993; 1.000) | 1.000  (0.999; 1.000) |
|  | age.hgt.bag | 1.000  (0.993; 1.000) | 1.000  (0.999; 1.000) | 1.000  (0.993; 1.000) | 1.000  (0.999; 1.000) |
|  | hgt.wgt.bag | 1.000  (0.993; 1.000) | 1.000  (0.999; 1.000) | 1.000  (0.993; 1.000) | 1.000  (0.999; 1.000) |
|  | maha.C | 0.054  (0.036; 0.076) | 1.000  (0.999; 1.000) | 0.967  (0.828; 0.999) | 0.897  (0.888; 0.905) |
|  | maha.MCD | 1.000  (0.993; 1.000) | 0.925  (0.917; 0.933) | 0.619  (0.585; 0.651) | 1.000  (0.999; 1.000) |
|  | maha.MVE | 1.000  (0.993; 1.000) | 0.954  (0.947; 0.960) | 0.724  (0.690; 0.756) | 1.000  (0.999; 1.000) |
|  | DDC | 1.000  (0.993; 1.000) | 0.993  (0.990; 0.995) | 0.942  (0.920; 0.960) | 1.000  (0.999; 1.000) |
| Swap last digits | SDS | 0.057  (0.039; 0.080) | 1.000  (0.999; 1.000) | 1.000  (0.888; 1.000) | 0.898  (0.889; 0.906) |
|  | box | 0.017  (0.008; 0.031) | 1.000  (0.999; 1.000) | 1.000  (0.664; 1.000) | 0.894  (0.885; 0.902) |
|  | age.hgt.bag | 0.274  (0.237; 0.314) | 1.000  (0.999; 1.000) | 1.000  (0.975; 1.000) | 0.919  (0.911; 0.927) |
|  | hgt.wgt.bag | 0.367  (0.326; 0.409) | 0.998  (0.996; 0.999) | 0.961  (0.925; 0.983) | 0.929  (0.921; 0.936) |
|  | maha.C | 0.154  (0.124; 0.187) | 0.999  (0.998; 1.000) | 0.965  (0.901; 0.993) | 0.907  (0.899; 0.915) |
|  | maha.MCD | 0.593  (0.550; 0.634) | 0.922  (0.914; 0.930) | 0.479  (0.441; 0.518) | 0.949  (0.942; 0.956) |
|  | maha.MVE | 0.546  (0.503; 0.589) | 0.967  (0.961; 0.972) | 0.664  (0.618; 0.708) | 0.946  (0.939; 0.953) |
|  | DDC | 0.567  (0.524; 0.609) | 0.977  (0.972; 0.981) | 0.748  (0.703; 0.790) | 0.949  (0.942; 0.955) |
| Add 40cm | SDS | 0.081  (0.060; 0.108) | 0.992  (0.989; 0.995) | 0.557  (0.441; 0.669) | 0.899  (0.890; 0.907) |
|  | box | 0.000  (0.000; 0.007) | 1.000  (0.999; 1.000) | - | 0.892  (0.883; 0.900) |
|  | age.hgt.bag | 0.285  (0.247; 0.325) | 1.000  (0.998; 1.000) | 0.987  (0.954; 0.998) | 0.920  (0.912; 0.928) |
|  | hgt.wgt.bag | 0.606  (0.563; 0.647) | 0.992  (0.989; 0.994) | 0.898  (0.863; 0.927) | 0.954  (0.948; 0.960) |
|  | maha.C | 0.009  (0.003; 0.021) | 0.999  (0.997; 1.000) | 0.455  (0.167; 0.766) | 0.893  (0.884; 0.901) |
|  | maha.MCD | 0.956  (0.935; 0.971) | 0.926  (0.918; 0.933) | 0.609  (0.575; 0.642) | 0.994  (0.991; 0.996) |
|  | maha.MVE | 0.959  (0.939; 0.974) | 0.954  (0.947; 0.960) | 0.716  (0.682; 0.749) | 0.995  (0.992; 0.997) |
|  | DDC | 0.722  (0.682; 0.760) | 0.977  (0.972; 0.981) | 0.789  (0.751; 0.825) | 0.967  (0.961; 0.972) |
| Sample from 1^s^t percentile | SDS | 0.006  (0.001; 0.016) | 1.000  (0.999; 1.000) | 0.750  (0.194; 0.994) | 0.893  (0.884; 0.901) |
|  | box | 0.000  (0.000; 0.007) | 1.000  (0.999; 1.000) | - | 0.892  (0.883; 0.900) |
|  | age.hgt.bag | 0.004  (0.000; 0.013) | 1.000  (0.999; 1.000) | 1.000  (0.158; 1.000) | 0.892  (0.883; 0.901) |
|  | hgt.wgt.bag | 0.009  (0.003; 0.021) | 0.998  (0.997; 0.999) | 0.417  (0.152; 0.723) | 0.893  (0.884; 0.901) |
|  | maha.C | 0.024  (0.013; 0.041) | 0.997  (0.995; 0.998) | 0.500  (0.299; 0.701) | 0.894  (0.885; 0.902) |
|  | maha.MCD | 0.289  (0.251; 0.329) | 0.927  (0.919; 0.935) | 0.325  (0.283; 0.369) | 0.915  (0.907; 0.923) |
|  | maha.MVE | 0.191  (0.158; 0.226) | 0.964  (0.958; 0.969) | 0.390  (0.331; 0.452) | 0.908  (0.899; 0.916) |
|  | DDC | 0.237  (0.202; 0.275) | 0.976  (0.971; 0.980) | 0.542  (0.477; 0.607) | 0.914  (0.905; 0.921) |

PPV=positive predictive value; NPV= negative predictive value

Supplement 3.3 ROC curves: computation of sensitivity and specificity at Youden threshold

| Prevalence of errors 2% |
| --- |
| 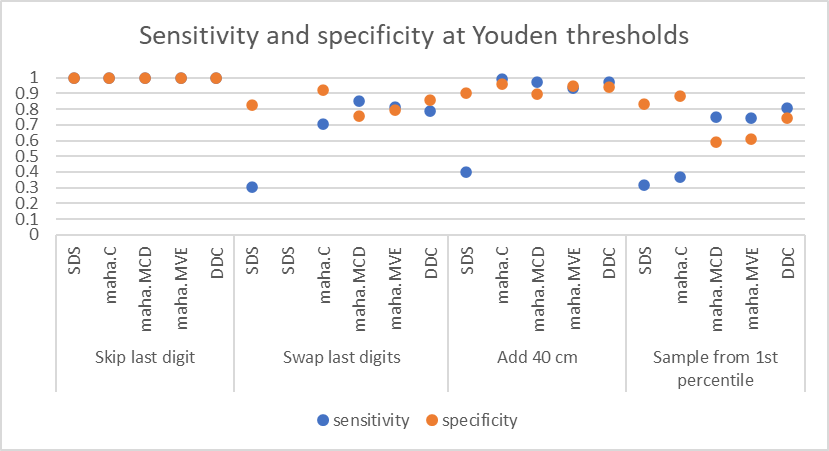 |
| Prevalence of errors 10% |
| 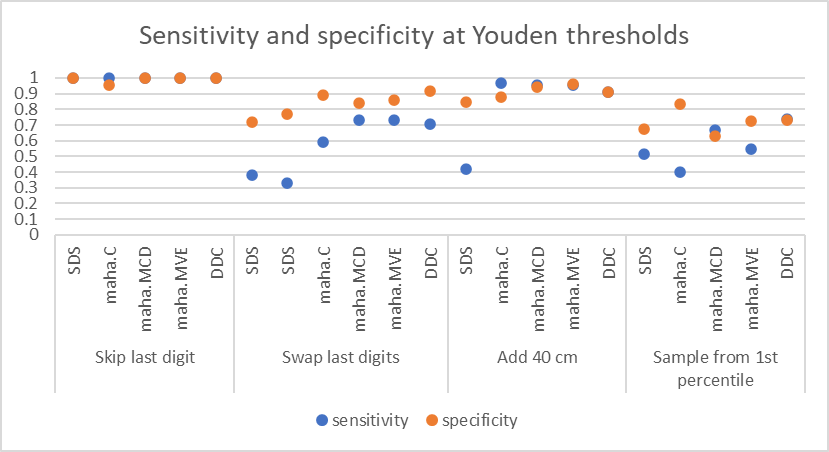 |

Note: for error pattern “swap last digits”, the Youden index was equivalent for two thresholds when we used the SDS method; for this reason, we reported levels of sensitivity and specificity at both thresholds

Supplement 3.4 ROC curves computation of sensitivity and specificity at Youden threshold (prevalence of errors 2%)

| **Error** | **Method** | **threshold** | **Sensitivity**  **95% CI** | **specificity**  **95% CI** |
| --- | --- | --- | --- | --- |
| Skip last digit | SDS | 2.889 | 1.000  (0.966; 1.000) | 1.000  (0.966; 1.000) |
|  | maha.C | 19.238 | 1.000  (0.966; 1.000) | 1.000  (0.966; 1.000) |
|  | maha.MCD | 182.789 | 1.000  (0.966; 1.000) | 1.000  (0.966; 1.000) |
|  | maha.MVE | 184.649 | 1.000  (0.966; 1.000) | 1.000  (0.966; 1.000) |
|  | DDC | 12.240 | 1.000  (0.966; 1.000) | 1.000  (0.966; 1.000) |
| Swap last digits | SDS | 1.188 | 0.306  (0.221; 0.402) | 0.828  (0.221; 0.402) |
|  | maha.C | 5.747 | 0.704  (0.608; 0.788) | 0.919  (0.608; 0.788) |
|  | maha.MCD | 6.782 | 0.852  (0.771; 0.913) | 0.754  (0.771; 0.913) |
|  | maha.MVE | 7.976 | 0.815  (0.729; 0.883) | 0.798  (0.729; 0.883) |
|  | DDC | 1.567 | 0.787  (0.698; 0.860) | 0.861  (0.698; 0.860) |
| Add 40 cm | SDS | 1.424 | 0.398  (0.305; 0.497) | 0.902  (0.305; 0.497) |
|  | maha.C | 7.546 | 0.991  (0.949; 1.000) | 0.960  (0.949; 1.000) |
|  | maha.MCD | 15.625 | 0.972  (0.921; 0.994) | 0.896  (0.921; 0.994) |
|  | maha.MVE | 22.152 | 0.935  (0.871; 0.974) | 0.947  (0.871; 0.974) |
|  | DDC | 2.287 | 0.972  (0.921; 0.994) | 0.943  (0.921; 0.994) |
| Sample from 1^st^ percentile | SDS | 0.249 | 0.315  (0.229; 0.411) | 0.834  (0.229; 0.411) |
|  | maha.C | 5.282 | 0.370  (0.279; 0.469) | 0.882  (0.279; 0.469) |
|  | maha.MCD | 3.899 | 0.750  (0.657; 0.828) | 0.594  (0.657; 0.828) |
|  | maha.MVE | 4.017 | 0.741  (0.648; 0.820) | 0.611  (0.648; 0.820) |
|  | DDC | 1.125 | 0.806  (0.718; 0.875) | 0.745  (0.718; 0.875) |

Supplement 3.5 ROC curves computation of sensitivity and specificity at Youden threshold (prevalence of errors 10%)

| **Error** | **Method** | **threshold** | **Sensitivity**  **95% CI** | **specificity**  **95% CI** |
| --- | --- | --- | --- | --- |
| Skip last digit | SDS | 2.373 | 1.000  (0.993; 1.000) | 1.000  (0.993; 1.000) |
|  | maha.C | 6.006 | 1.000  (0.993; 1.000) | 0.958  (0.993; 1.000) |
|  | maha.MCD | 154.878 | 1.000  (0.993; 1.000) | 1.000  (0.993; 1.000) |
|  | maha.MVE | 129.693 | 1.000  (0.993; 1.000) | 1.000  (0.993; 1.000) |
|  | DDC | 9.477 | 1.000  (0.993; 1.000) | 1.000  (0.993; 1.000) |
| Swap last digits | SDS | 0.982 | 0.381  (0.340; 0.424) | 0.721  (0.340; 0.424) |
|  | SDS | 1.070 | 0.331  (0.292; 0.373) | 0.771  (0.292; 0.373) |
|  | maha.C | 4.332 | 0.589  (0.546; 0.631) | 0.891  (0.546; 0.631) |
|  | maha.MCD | 9.204 | 0.730  (0.690; 0.767) | 0.838  (0.690; 0.767) |
|  | maha.MVE | 7.348 | 0.730  (0.690; 0.767) | 0.857  (0.690; 0.767) |
|  | DDC | 1.724 | 0.706  (0.665; 0.744) | 0.917  (0.665; 0.744) |
| Add 40 cm | SDS | 1.364 | 0.420  (0.378; 0.463) | 0.845  (0.378; 0.463) |
|  | maha.C | 4.374 | 0.969  (0.950; 0.982) | 0.879  (0.950; 0.982) |
|  | maha.MCD | 18.438 | 0.952  (0.930; 0.968) | 0.939  (0.930; 0.968) |
|  | maha.MVE | 17.651 | 0.954  (0.932; 0.970) | 0.963  (0.932; 0.970) |
|  | DDC | 1.765 | 0.909  (0.882; 0.932) | 0.912  (0.882; 0.932) |
| Sample from 1^st^ percentile | SDS | 0.475 | 0.513  (0.470; 0.556) | 0.676  (0.470; 0.556) |
|  | maha.C | 4.302 | 0.402  (0.360; 0.445) | 0.832  (0.360; 0.445) |
|  | maha.MCD | 3.933 | 0.669  (0.627; 0.708) | 0.627  (0.627; 0.708) |
|  | maha.MVE | 4.687 | 0.546  (0.503; 0.589) | 0.725  (0.503; 0.589) |
|  | DDC | 0.992 | 0.741  (0.702; 0.777) | 0.730  (0.702; 0.777) |

Supplement 3.6 Positive Likelihood Ratios (LR+) across scenarios

|  | | **Prevalence of errors** | |
| --- | --- | --- | --- |
|  |  | **2%** | **10%** |
| **Error** | **method** | **LR+ (95%CI)** | **LR+ (95%CI)** |
| Skip last digit | SDS | - | - |
|  | box | - | - |
|  | age.hgt.bag | - | - |
|  | hgt.wgt.bag | 349.4  (207.1; 589.6) | - |
|  | maha.C | 2446.0  (611.9; 9777.4) | 239.5  (32.7; 1754.8) |
|  | maha.MCD | 10.0  (9.2; 10.8) | 13.4  (12.1; 14.9) |
|  | maha.MVE | 11.1  (10.1; 12.1) | 21.7  (18.9; 24.7) |
|  | DDC | 28.3  (24.4; 32.7) | 135.2  (96.2; 189.9) |
| Swap last digits | SDS | 226.5  (26.7; 1922.2) | - |
|  | box | - | - |
|  | age.hgt.bag | 2083.6  (290.0; 14970.3) | - |
|  | hgt.wgt.bag | 113.2  (67.8; 189.0) | 204.4  (101.4; 412.1) |
|  | maha.C | 123.8  (70.7; 216.7) | 228.5  (72.5; 720.5) |
|  | maha.MCD | 6.7  (5.7; 7.8) | 7.6  (6.7; 8.6) |
|  | maha.MVE | 7.4  (6.3; 8.7) | 16.4  (13.7; 19.5) |
|  | DDC | 17.5  (14.4; 21.4) | 24.5  (20.0; 30.1) |
| Add 40cm | SDS | 181.2  (38.9; 843.2) | 10.4  (6.7; 16.0) |
|  | box | - | - |
|  | age.hgt.bag | 3578.4  (502.5; 25483.0) | 636.0  (158.1; 2558.4) |
|  | hgt.wgt.bag | 234.0  (146.7; 373.4) | 73.0  (52.6; 101.3) |
|  | maha.C | 170.5  (103.5; 281.0) | 6.9  (2.1; 22.5) |
|  | maha.MCD | 9.7  (8.8; 10.6) | 12.9  (11.6; 14.3) |
|  | maha.MVE | 10.7  (9.7; 11.7) | 20.9  (18.2; 23.9) |
|  | DDC | 25.1  (21.6; 29.2) | 31.0  (25.4; 37.7) |
| Sample from 1^s^t percentile | SDS | - | 24.8  (2.6; 237.8) |
|  | box | - | - |
|  | age.hgt.bag | 90.6  (8.3; 991.5) | - |
|  | hgt.wgt.bag | 14.2  (5.3; 37.9) | 5.9  (1.9; 18.5) |
|  | maha.C | 15.1  (6.6; 34.8) | 8.3  (3.8; 17.7) |
|  | maha.MCD | 2.8  (2.1; 3.9) | 4.0  (3.4; 4.7) |
|  | maha.MVE | 3.2  (2.4; 4.4) | 5.3  (4.2; 6.7) |
|  | DDC | 7.5  (5.3; 10.4) | 9.8  (7.7; 12.4) |

The LR+ is the ratio between the true positivity rate (sensitivity) and the false positivity rate (1-specificity), therefore it is the probability of an error being flagged as such divided by the probability of a genuine value being flagged as an error. It can be used to compute a post-test probability, i.e. the probability of a value being flagged as an error after the test has been performed. The higher the LR+, the higher the post-test probability, but the threshold beyond which it is considered a meaningful change from pre-test to post-test probability depends heavily on the context. In our simulations, there wasn’t a clear pattern of LR+ across scenarios: as shownhere, LR+ ranged from around 4 (95%CI: 3.4; 4.7 for error pattern “sample from 1st percentile” detected by Mahalanobis distance using MCD) to over 600 (95%CI: 158.1; 2558.4 for error “add 40 cm” detected by bagplots). The extremely high values for LR+ are due to the high values for specificity, which make denominators for LR+ very small.
